# Editorial for the Special Issue “Molecular and Cellular Mechanisms of CVD: Focus on Atherosclerosis (Volume II)”

**DOI:** 10.3390/biomedicines13092047

**Published:** 2025-08-22

**Authors:** Nikita G. Nikiforov

**Affiliations:** 1 Laboratory of Angiopathology, The Institute of General Pathology and Pathophysiology, 125315 Moscow, Russia; nikiforov.mipt@googlemail.com; 2 Center for Precision Genome Editing and Genetic Technologies for Biomedicine, Institute of Gene Biology, Russian Academy of Sciences, 119334 Moscow, Russia; 3 Engelhardt Institute of Molecular Biology, Russian Academy of Sciences, 119991 Moscow, Russia

This Special Issue, “Molecular and Cellular Mechanisms of CVD: Focus on Atherosclerosis (Volume II)”, presents a collection of original and review articles exploring the complex biological mechanisms underlying atherosclerosis and its associated cardiovascular complications. The studies cover novel biomarkers, genetic predispositions, metabolic disorders, and therapeutic approaches, providing deeper insights into disease pathogenesis and potential treatment strategies.

## 1. Inflammation, Thrombosis, and Vascular Dysfunction in Atherosclerosis

The interplay between inflammation and thrombosis is a key aspect of atherosclerosis pathogenesis. The authors of [1] investigated the relationship between inflammatory and coagulation markers and cognitive/motor impairments in patients with atrial fibrillation (AF). AF is a cardiac arrhythmia in which the upper chambers of the heart (atria) fail to contract effectively, instead quivering or “fibrillating”, leading to reduced blood pumping efficiency and an increased risk of thrombus formation. A prothrombotic state—marked by elevated von Willebrand factor (VWF) levels and prolonged clot lysis time—was associated with delayed response times in cognitive tests. Elevated interleukin-8 (IL-8) levels also correlated with reduced physical performance. These biomarkers may help to identify AF patients at higher risk of developing cognitive and motor impairments. This is particularly important given the current inability of clinicians to predict which AF patients will develop dementia or mobility decline.

In [2], the authors examined VWF dynamics in patients with stable coronary artery disease (CAD). The impaired collagen-binding activity of VWF (VWF:CB) alongside enhanced platelet adhesion suggests the potential role of dysfunctional VWF–collagen–platelet interactions in the progression of CAD.

The authors of [3] demonstrated that prolonged cardiopulmonary bypass during cardiac surgery induces inflammation via the activation of the MMP9 enzyme, leading to glypican-1 shedding and endothelial dysfunction. These findings underscore the critical role of inflammation in both acute vascular injury and chronic cerebrovascular complications.

## 2. Genetic Factors in Atherosclerosis

Genetic predisposition plays a major role in the development of atherosclerosis. In [4], the gene C11orf58 (encoding the Hero20 protein) is identified as a novel factor influencing the risk of ischemic stroke, particularly in smokers and individuals with low fruit and vegetable intake. The chaperone-like functions of Hero20 may protect against protein aggregation and disruptions in cholesterol metabolism, linking genetic variation to oxidative stress and vascular remodeling.

Mitochondrial dysfunction also contributes to atherosclerosis, as shown in [5], where specific mitochondrial DNA mutations are associated with carotid plaque formation. The overall mutational burden of the mitochondrial genome may serve as a prognostic marker, highlighting the importance of mitochondrial health in CVD.

## 3. Metabolic and Lipid Dysregulation

Dyslipidemia is a central feature of atherosclerosis. In [6], differences in lipid profiles were identified in patients with brachiocephalic artery atherosclerosis. Alterations in saturated and unsaturated fatty acid levels, including omega-3 and omega-6 fatty acids, underscore the diagnostic potential of lipidomics.

Study [7] explored the therapeutic potential of fucoidan—a marine polysaccharide that enhances cholesterol efflux by upregulating ABCA1 expression and modulating scavenger receptors (SR-AI, CD36)—thus reducing lipid accumulation in macrophages. This positions fucoidan as a promising atheroprotective agent.

Metabolic dysfunction also plays a major role in pathogenesis, as discussed in [8], which addresses energy metabolism in heart failure. The balance between ATP synthesis and consumption, regulated via AMPK and PGC-1α, is critical for cardiac function, and its disruption exacerbates CVD progression. Article [9] expands this concept to immunometabolism, showing how the metabolic reprogramming of T cells (glycolysis vs. oxidative phosphorylation) determines their pro- or anti-atherogenic properties. Th1/Th17 cells, which rely on glycolysis, promote inflammation, while Treg cells utilize fatty acid oxidation—making metabolic pathways attractive therapeutic targets.

Age-related changes in the immune system are examined in [10], highlighting thymic involution and its impact on T cell maturation. Age-dependent replacement of the thymus with adipose tissue and the dysregulation of adipokines (e.g., leptin) may exacerbate inflammation and atherosclerosis, linking immunosenescence to CVD progression.

## 4. Conclusions

This Special Issue brings together diverse yet interconnected areas of research—genetic, metabolic, inflammatory, and thrombotic—that collectively shape the development of atherosclerosis. The findings highlight new biomarkers (e.g., VWF:CB and mtDNA mutations), genetic risk factors (C11orf58), and potential therapeutic targets (fucoidan and metabolic modulators). Looking forward, personalized approaches targeting specific molecular pathways may improve CVD treatment, emphasizing the need for continued translational research. I thank the authors for their valuable contributions and hope that this collection of articles will inspire further exploration of the molecular and cellular mechanisms of atherosclerosis, paving the way for innovative therapeutic strategies.
